# Characterization and genomic analysis of the first Oceanospirillum phage, vB_OliS_GJ44, representing a novel siphoviral cluster

**DOI:** 10.1186/s12864-021-07978-4

**Published:** 2021-09-20

**Authors:** Wenjing Zhang, Yantao Liang, Kaiyang Zheng, Chengxiang Gu, Yundan Liu, Ziyue Wang, Xinran Zhang, Hongbing Shao, Yong Jiang, Cui Guo, Hui He, Hualong Wang, Yeong Yik Sung, Wen Jye Mok, Yuzhong Zhang, Andrew McMinn, Min Wang

**Affiliations:** 1 College of Marine Life Sciences, Frontiers Science Center for Deep Ocean Multispheres and Earth System, Institute of Evolution and Marine Biodiversity, Ocean University of China, Qingdao, 266003 China; 2 UMT-OUC Joint Centre for Marine Studies, Qingdao, 266003 China; 3 Institute of Marine Biotechnology, Universiti Malaysia Terengganu (UMT), 21030 Kuala Nerus, Malaysia; 4 Shangdong University, Qingdao, 266000 China; 5 Institute for Marine and Antarctic Studies, University of Tasmania, Hobart, Tasmania 7001 Australia; 6 The Affiliated Hospital of Qingdao University, Qingdao, 266000 China

**Keywords:** *Oceanospirillum*, Phage vB_OliS_GJ44, *Oceanospirivirus*, Genomics, Metagenomics, Tail-related genes, Recombination

## Abstract

**Background:**

Marine bacteriophages play key roles in the community structure of microorganisms, biogeochemical cycles, and the mediation of genetic diversity through horizontal gene transfer. Recently, traditional isolation methods, complemented by high-throughput sequencing metagenomics technology, have greatly increased our understanding of the diversity of bacteriophages. *Oceanospirillum,* within the order *Oceanospirillales*, are important symbiotic marine bacteria associated with hydrocarbon degradation and algal blooms, especially in polar regions. However, until now there has been no isolate of an Oceanospirillum bacteriophage, and so details of their metagenome has remained unknown.

**Results:**

Here, we reported the first Oceanospirillum phage, vB_OliS_GJ44, which was assembled into a 33,786 bp linear dsDNA genome, which includes abundant tail-related and recombinant proteins. The recombinant module was highly adapted to the host, according to the tetranucleotides correlations. Genomic and morphological analyses identified vB_OliS_GJ44 as a siphovirus, however, due to the distant evolutionary relationship with any other known siphovirus, it is proposed that this virus could be classified as the type phage of a new *Oceanospirivirus* genus within the *Siphoviridae* family. vB_OliS_GJ44 showed synteny with six uncultured phages, which supports its representation in uncultured environmental viral contigs from metagenomics. Homologs of several vB_OliS_GJ44 genes have mostly been found in marine metagenomes, suggesting the prevalence of this phage genus in the oceans.

**Conclusions:**

These results describe the first Oceanospirillum phage, vB_OliS_GJ44, that represents a novel viral cluster and exhibits interesting genetic features related to phage–host interactions and evolution. Thus, we propose a new viral genus *Oceanospirivirus* within the *Siphoviridae* family to reconcile this cluster, with vB_OliS_GJ44 as a representative member.

**Supplementary Information:**

The online version contains supplementary material available at 10.1186/s12864-021-07978-4.

## Background

From the ocean surface to the hadal zones and from the Arctic to the Antarctic, viruses are the most abundant and diverse life forms in the ocean [1, 2]. They control the microbial community through infection and lysis of their hosts, which promote biogeochemical cycling through the “viral shunt” and “viral shuttle” [3]. Viruses also mediate the horizontal gene transfer and the evolution of their hosts and contribute to marine carbon sequestration through the “biological pump” and “microbial carbon pump” [4–6]. However, more than 90% of the viral population remains unknown [7]. Thus, an increase in phage identification will promote a better understanding of their evolution and their effects on microbial communities and biogeochemical cycles.

*Oceanospirillum* is the type genus of the family *Oceanospirillaceae*, in the order *Oceanospirillales* of the class *Gammaproteobacteria*. Members of this family have often been found in oil-contaminated habitats [1, 8–10], and are well known for their ability to degrade petroleum hydrocarbons [11]. They are also abundant in the Mariana Trench, suggesting potentially important roles in extreme environments [12]. Currently, six *Oceanospirillum* species have been identified from habitats including coastal areas, sediments, the deep-sea, putrid infusions of marine mussels and especially from oil-contaminated environments [13–17]. Despite the ecological importance of this bacteria lineage, our knowledge about the viruses infecting *Oceanospirillaceae* is quite few. Currently, only six phages infecting *Oceanospirillaceae* have been isolated so far, including five infecting *Marinomonas* and one infecting *Nitrincola*. Phages infecting other genera of *Oceanospirillum* have yet not been isolated*.*

In this study, we isolated and characterized the first bacteriophage infecting *Oceanospirillum*, vB_OliS_GJ44. It was found to possess novel genomic features and represented a novel siphoviral cluster. Combined with the eight environmental viral contigs from metagenomics, this study helps fill the gap in our understanding of the isolation, genomic and evolutionary development of *Oceanospirillum* bacteriophages and provides new insights into the interactions between hosts and bacteriophages for these important marine hydrocarbon-degrading microbial populations.

## Materials and methods

### Isolation of host *Oceanospirillum* sp. ZY01 and phage vB_OliS_GJ44

*Oceanospirillum* sp. ZY01 and its phage vB_OliS_GJ44 were both isolated from surface water samples in the Yellow Sea (35°23′59.582″N, 119°34′7.158″E) in October, 2019. 2216E media (peptone 5 wt.%, yeast extract 1 wt.%) dissolved in artificial seawater (Sigma) was used to culture and propagate the host. The host was able to be grown efficiently in shake cultivation at 28 °C and 120 rpm.

To obtain a concentrated sample of the phage, 50 L of coastal water was concentrated to 10 ml by tangential flow filtration with 50-kDa and 30-kDa cartridges, (Pellicon® XL Cassette, Biomax® 50 kDa; polyethersulfone, Millipore Corporation, Billerica, MA, USA), after passing through a 0.2-μm membrane filter (Isopore™ 0.2 μm GTTP; Merck, Ireland) [18]. A PE centrifuge tube was used to retain the concentrated viral stock, which was then stored in the dark place at 4 °C.

The double-layer plating method was used to isolate the phage. Briefly, 200 μl of concentrated viral stock was mixed with the host culture (approximately 10-h) and incubated for 20-min, allowing the absorption of the phages at room temperature. Then, 4 ml of the semi-solid culture at 45 °C was added into the mixture, pouring onto the plate after vortex. Plates were cultivated at 27 °C for 24-h and visible plaques were formed in the double layer culture.

### Purification and concentration of vB_OliS_GJ44

A single plaque was picked, placed in SM buffer and shaken for 3-h at 120 rpm to dissociate the viral particles from the agar. The mixture was passed through a 0.22 μm membrane filter and allowed to infect the host, as described above. This step was repeated five times to obtain purified viral stock.

To concentrate the viral stock, 5 ml of purified viral stock was incubated with 50 ml of the exponentially growing host at 28 °C for 12-h. The mixture was filtered through a 0.22-μm membrane filter to harvest phages particles, and PFU (plaque-forming unit) was counted by flow cytometry to assess the efficiency of propagation.

The lysate was concentrated from 50 ml to 2 ml using an ultrafilter (Milipore® Amicon Ultra-15) under 5000 g. And the concentrated and purified viral stock was stored in the dark place at 4 °C.

### Morphological identification, host range test and one-step growth of vB_OliS_GJ44

The morphology of vB_OliS_GJ44 was characterized by transmission electron microscopy (TEM) using established protocols of the negative staining method [19]. A drop of 20 μl concentrated, purified viral stock (~ 10^9^ PFU/ml) was placed on the copper net, stained with 2 wt.% phosphotungstic acids (pH 7.5) for 5 min, and then observed under the TEM (JEOLJEM-1200EX, Japan) at 100 KV.

The host range test was performed using the double-layer plating method on 35 *Oceanospirillales* strains. In summary, different bacterial cultures were mixed with a series of viruses in multiples according to the optimal multiplicity of infection (MOI); the mixture was then spread on a soft agar layer. Plaque formation was observed after incubating overnight at 28 °C.

The one-step growth assay was conducted following Sillankorva S. et al. [20]. Briefly, the exponentially growing host culture (~ 10^8^ CFU/L) was mixed with vB_OliS_GJ44 stock under the MOI 0.01 and incubated for 30 min. Then, the mixture was centrifuged (6000 g) for 5 min and the supernatant discarded to remove unabsorbed phages, the pellet was then resuspended in 1 ml of 2216E medium. This step was repeated three times and the sample was then transferred to 300 ml 2216E medium and shaken at 28 °C for 180-min. Sampling was conducted throughout the incubation at 10-min intervals. Each sample was immediately fixed with glutaraldehyde (final concentration: 0.5%), flash-frozen in the liquid nitrogen and stored at − 80 °C prior to analysis. Flow cytometry was used to count the viral particles of each sample, as described above (water bath for 10 min at 80 °C). Three parallel tests were conducted for this assay.

### The phylogeny of *Oceanospirillum* sp. ZY01

A total of 121 *Oceanospirillaceae* reference sequences of 16S rRNA genes, including the host strain *Oceanospirillum* sp. ZY01, were retrieved from GenBank and aligned by mafft [21] using G-INS-1 of strategy with 1000 iterations. The phylogenic tree was calculated from multiple sequence alignments using IQ-tree2 [22], applying GTR + F + R4 as the suggested DNA model with 1000 iterations of bootstrap. The tree was visualized by iTOL v4 [23].

### Genome sequencing and annotation of vB_OliS_GJ44

Sequencing was performed by Shanghai Biozeron Biotechnology Co., Ltd. (Shanghai, China.). The high-quality DNA sample was used to construct an Illumina pair-end library and then used for Illumina NovaSeq 6000 sequencing. The raw paired-end reads were trimmed and quality controlled by Trimmomatic (v. 0.3.6) with parameters: SLIDINGWINDOW:4:15, MINLEN:75 [24]. ABySS was used to assemble the viral genome after the quality control processes, multiple-Kmer parameters were chosen to obtain the optimal assembly results [25]. GapCloser software was subsequently applied to fill in the remaining local inner gaps and to correct the single base polymorphism for the final assembly and for further analysis [26].

Coding DNA sequences of phage vB_OliS_GJ44 were predicted using GeneMarkS [27], RAST [28], and Glimmer [29]. All open reading frames (ORFs) were annotated by BLASTp and Position-Specific Iterated BLAST (PSI-BLAST), against the nonredundant proteins (NR) NCBI database (*e*-value was set at 1e-5, identity > 30%). PSI-BLAST was used to identify the putative proteins in the structural gene cluster of the phage (non-default parameters: num_iterations 1000, *e*-value <1e-5, query coverage (qcov) > 50%). The InterPro database [30], the Conserved Domain Database suite (CDD/SPARCLE) [31], the UniProtKB database [32], and the HHpred server [33] were used to detect the conserved domain in every ORF. Possible inconsistencies, produced by different prediction and annotation tools, were checked manually. Easyfig v2.2.2 was used for genome visualization and tRNAscan-SE was used for tRNA gene detection [34, 35].

Moreover, GC skew analysis was performed on Webskew, which is the online version of Genskew (https://genskew.csb.univie.ac.at/webskew).

### Phylogenetic analysis of vB_OliS_GJ44

The major capsid protein (MCP) was selected as the hallmark protein to be identified by BLASTp from the NR database. A total of 50 best hit sequences were selected and aligned using MUSCLE [36], with *e*- value 1E-150, 99% coverage and 65% identity cutoff. A maximum-likelihood phylogenetic tree was generated using MEGA v10 [37] and visualized with iTOL v4 [23]. Another phylogenetic tree constructed for the terminase large subunit (TerL) was undertaken in the same way as for MCP.

A proteomic tree based on the similarities of the whole genome was generated using VIPTree (https://www.genome.jp/viptree/) [38]. Each encoding nucleic sequences as a query were searched against the Virus-Host DB using tBLASTx. All viral sequences in Virus-Host DB were selected to generate a circular tree. The 461 related phages in the circular tree were automatically selected by VIPtree according to genomic similarity scores (*S*_*G*_) larger than 0.05, then used to generate a more accurate phylogenetic tree with vB_OliS_GJ44.

Three conserved genes (MCP, TerL, and portal protein) were selected as hallmark proteins to build a polygenic phylogenetic tree of the extended vB_OliS_GJ44. Homologous proteins of three hallmark proteins were identified using Diamond blastp (v0.9.4.105), with *e*-value 1 × 10^− 5^ and 85% qcov cut-off. These sequences were retrieved and aligned using MUSCLE [36]. Sixty-seven viral genomes with at least two of the three homologous hallmark proteins were selected. Gap were removed from the alignment with trimal [39] and connected with seqkit [40]. A maximum-likelihood phylogenetic tree was then calculated based on the concatenated alignment of all three proteins with IQ-tree2 with ultrafast bootstrap 1000 and VT + F + R4 as suggested by the model test as the best-fit substitution model [22]. The phylogenetic tree was visualized with iTOL v4 [23].

### Phage vB_OliS_GJ44 homologs in IMG/VR

To expand the phage vB_OliS_GJ44 group, each coding sequence was queried against the IMG/VR [41] database using tBLASTx to search for homologous proteins and to map the contig ID (threshold: *e*-value <1e-5, idendity > 20, −max_target_seqs 1). Virus contigs with more than five homologous sequences were selected and removed the low-quality contigs according the information of IMG/VR [41]. Finally, 25 uncultivated high-quality virus contigs and 13 isolated sequences were selected as references, of which eight Brucella phages appeared as an outgroup. All these 27 sequences and the vB_OliS_GJ44 genome were used to construct the whole-genome phylogenetic tree using VIPTree [38].

Average nucleotide sequence identity was calculated by OAT software, which used the orthogonal method to determine the overall similarity between the two genomic sequences [42].

### Environmental distribution of phage vB_OliS_GJ44

The relative abundance of vB_OliS_GJ44 was assessed through three marine viral metagenomic datasets, including Pacific Ocean Virome (POV) [43], Global Ocean Sampling (GOS) [44] (available at CAMERA (http://camera.calit2.net), and Malaspina (available at www.pangaea.de) viral metagenomes [45]. A total 67 viruses were retrieved from the three datasets and eciprocal best-hit BLASTp (RBB), as applied by Zhao et al. [46], was used to avoid potential false-positive homologies. To identify the homologs of vB_OliS_GJ44 proteins, BLAST nucleic acid libraries were built from each virome, and proteins of vB_OliS_GJ44 were compared against libraries by tBLASTn (non-default parameters -max_target_seqs 10,000,000, −max_hsps 1, −seg no, −outfmt 6). Then, subjects matched in the last step were extracted and were compared against the proteins of vB_OliS_GJ44 by BLASTx (non-default parameter: -max_target_seqs 10,000,000, −max_hsps 1, −seg no, −outfmt 6) Reciprocal best hits were retained as the final result. The relative abundance of each ORF was calculated by two normalizations, the total number of reads in each metagenome and the number of amino acids of each ORF.

### Tetranucleotides (tetra) correlations analysis

Thirty-four fragments were sliced from the nucleic acid sequence of vB_OliS_GJ44 (10 kbp for window size, 1 kbp for step size), the sequence was extended to both sides to avoid the bias of uneven slicing. Thus, each 1 kbp of the genome could be presented by a corresponding 10 kbp fragment. Two hundred fifty-six combinations of tetra frequency (from “AAAA” to “TTTT”) were calculated for each fragment, and normalized by z-scoring. The Pearson’s correlation coefficient was calculated from either the array of each fragment and that of the host genome as a whole, or the array of each fragment and that of the viral genome as a whole [47].

## Results and discussion

### Isolation, morphology, host range, and one-step growth

**Fig. 1:**
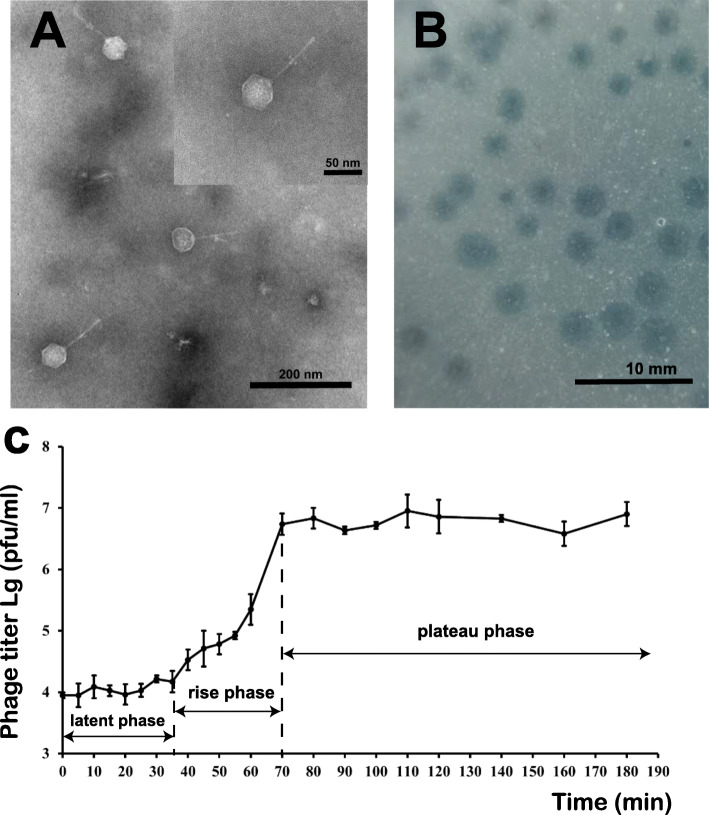
Morphology and biological properties of phage vB_OliS_GJ44. **A** Electron micrographs of Oceanospirillum phage vB_OliS_GJ44. vB_OliS_GJ44 lysate was stained with 4% uranyl acetate on a copper grid and viewed with a Philips/FEI transmission electron microscope. **B** Phage plaques formed in double-layer agar plate after culturing 24 h. **C** Increase in phage titers during one-step growth. The data shown are averages from triplicate experiments, and error bars indicate SDs

The bacteriophage vB_OliS_GJ44, infecting *Oceanospirillum* sp. ZY01 (accession: MW547060), was isolated from a surface seawater sample from the Yellow Sea; this is the first reported phage infecting this genus. Infection by vB_OliS_GJ44 formed clear and round (2–3 mm diameter average) plaques. The center of the plaque was more transparent than the rest (Fig. 1B). TEM results show that the vB_OliS_GJ44 viral particle possesses a siphoviral morphology. Measurements of 20 vB_OliS_GJ44 phage particles showed it had an icosahedral head, with an average diameter of 47 nm, and a long non-contractile tail, with an average length of 76 nm (Fig. 1A). The graph of TEM showed an interesting and special structure in the middle of the tail, which is similar to a tail filament. To the best of our knowledge, vB_OliS_GJ44 is the first phage where the tail filament is located in other positions of the tail.

The host cross-infection experiment showed that phage vB_OliS_GJ44 has a narrow host range. Of the 35 strains tested, it was found to only infect four strains of *Oceanospirillum scanctuarii* OLL623, OSL14, OSX334, and its propagating host bacterium ZY01 (Table 1). It could not infect *Oceanospirillum scanctuarii* 1A14960, even though they have a close evolutionarily relationship. This result is consistent with our understanding of the species specificity of siphoviruses. The one-step growth curve of phage vB_OliS_GJ44 showed the latent period was approximately 35 min and reached a growth plateau after 70 min. The burst size was approximately 107 viral particles released from each cell (Fig. 1C).

**Table 1:** Host range analysis of Oceanospirillum phage vB_OliS_GJ44

Species/strain	Susceptibility	Source
*Cobetia amphilecti* 10–4-4	–	a
*Cobetia amphilecti* 10–5-1	–	a
*Cobetia amphilecti* 432c	–	a
*Cobetia amphilecti* 587	–	a
*Cobetia crustatorum* 432e	–	a
*Marinobacterium* sp. 08XMAC-12	–	b
*Marinobacterium stanieri* LJ-7-3	–	a
*Marinobacterium stanieri* NH33	–	a
*Marinomonas arenicola*	–	b
*Marinomonas arenicola* KMM 3893	–	b
*Marinomonas arenicola* LPB0063	–	b
*Marinomonas arenicola* NH722a	–	a
*Marinomonas arenicola* NQ451f	–	a
*Marinomonas dokdonensis* DSW10–10	–	a
*Marinomonas foliarum* NH742c	–	a
*Marinomonas hwangdonensis* D64	–	a
*Marinomonas polaris* CK13	–	b
*Marinomonas polaris* T27	–	b
*Marinomonas primoryensis* K-6-2-3	–	a
*Marinomonas primoryensis* NQ142f	–	a
*Marinomonas profundimaris* D104	–	b
*Marinomonas ushuaiensis* U1	–	b
*Marinospirillum perlucidum* F3212	–	b
*Nitrincola schmidtii* R4–8	–	b
*Oceaniserpentilla haliotis*	–	b
*Oceaniserpentilla haliotis* DSM 19503	–	b
*Oceanospirillum linum* MCCC1F01216	–	a
*Oceanospirillum maris* NQ142d	–	a
*Oceanospirillum scanctuarii* 1A14960	–	a
*Oceanospirillum scanctuarii* OLL623	+	a
*Oceanospirillum scanctuarii* OSL14	+	a
*Oceanospirillum scanctuarii* OSX334	+	a
*Oceanospirillum* sp. ZY01	+	c
*Oleibacter marinus* B-675	–	a
*Oleispira* sp. DJHH37	–	b

^a^Bacterial strains kindly provided by Dr. Yuzhong Zhang, Key Laboratory of Microbiology, Shandong University

^b^Bacterial strains kindly provided by Dr. Qiliang Lai, Marine Culture Collection of China

^c^Propagating host bacterium in this study, isolated from the Yellow Sea, China

The 16S rRNA sequences of each strain used in the host range test have been provided in Additional file 2: 16S rRNA gene sequences of the bacteria used in host range test.fasta (51 kb)

### The phylogeny of *Oceanospirillum* sp. ZY01

**Fig. 2:**
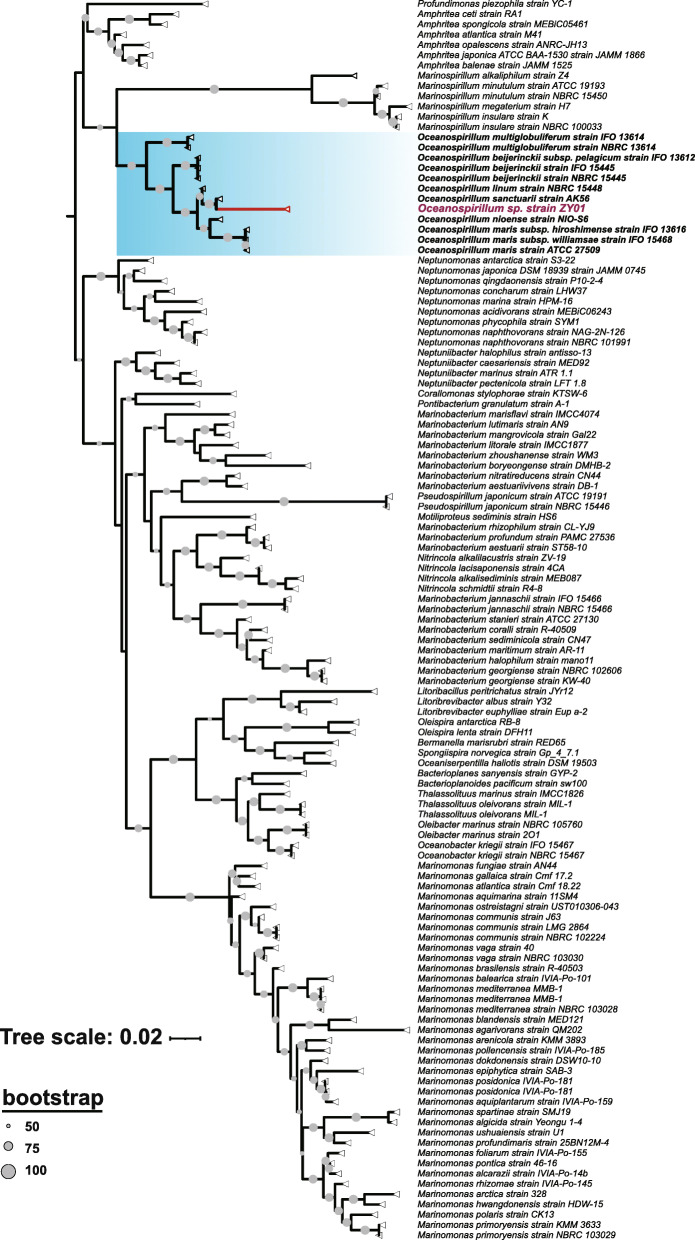
The phylogenic tree based on the 16S rRNA gene of *Oceanospirillum* sp*.* ZY01 and other 120 reference 16S rRNA gene sequences of *Oceanospirillaceae*

From the phylogenic tree based on the 16S rRNA gene of *Oceanospirillum* sp. ZY01 and other 120 reference sequences of *Oceanospirillaceae* (Fig. 2), *Oceanospirillum* sp. ZY01 was the most closely related to *Oceanospirillum sanctuarii* strain AK56, but had farther distance length from the branch root (*n* = 0.047) than *Oceanospirillum sanctuarii* strain AK56 (*n* = 0.002), indicating that *O.* sp. ZY01 might represent a novel variant of *Oceanospirillum sanctuarii*.

### Genomic features of Phage vB_OliS_GJ44

**Fig. 3:**
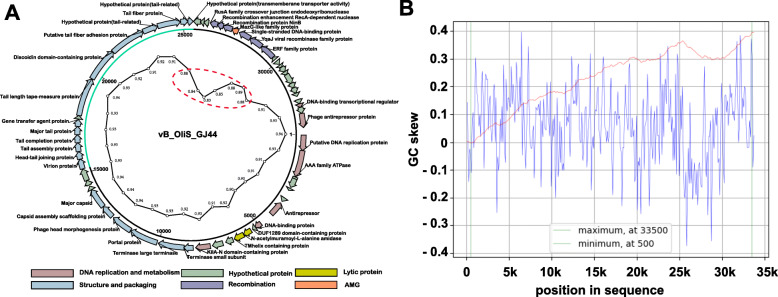
**A** Circularized genome map of vB_OliS_GJ44. The outer circle represents genes. Putative functional categories were defined according to annotation and are represented by different colors. The second circle shows the length of the genome, the green arc represents the length of the tail-related genes, and the third circle is a tetranucleotides correlation. The weaker correlations are circled by a red ellipse. **B** Cumulative GC skew analysis of the phage genome sequence. The global minimum and maximum are displayed in the cumulative graph were calculated by using a window size of 1,00 bp and a step size of 100 bp. The GC-skew and the cumulative GC-skew are represented by blue and red lines, respectively. The minimum and maximum of a GC-skew can be used to predict the origin of replication (500 nt) and the terminus location (33,500 nt)

According to the sequencing and assembly results, vB_OliS_GJ44 had a 33,786-bp linear dsDNA genome with a GC content of 48.8%. No tRNA was found in the genome. The genome had a 92% encoding rate consisting of 60 predicted ORFs. There were 24 coding regions (40%) that did not match any homologous sequence under the restriction of *e*-value <1e-5 in all 60 coding DNA sequences (CDS). Among the remaining 36 CDS that matched homologous sequences, 32 identified specific functions, and 4 matched homologous sequences with proteins of unknown function. The 36 ORFs could be classified into six different modules: 19 ORFs for phage structure and packing proteins, seven for DNA replication and metabolism, six for recombination, two for lysis, and one auxiliary metabolic gene (AMG). The remaining ORFs were all classified into hypothetical proteins. Forty-eight genes are located on the sense strand, accounting for 80% of the total coding genes. There were few genes on the antisense strand, only twelve, eleven of which were continuous (ORF 38 - ORF 48), including all six recombination genes. In contrast, there were many and various genes on the sense strand (Fig. 3A, Additional file 1: Table S1). The cumulative GC skew analyses was performed in order to determine the origin and terminus of replication of the phage genome. The results (Fig. 3B) indicate that the origin of replication is at the position 500 nt, and a replication terminus could be located at the region 33,500 nt. Two inflection points were identified at above regions, indicating an asymmetric base composition, which are the lowest at the origin and the highest at the terminus [48]. The annotation of genome showed that the first gene encoded a replication protein (20–766 nt), which provided additional support to the origin of replication (Additional file 1: Table S1).

### Genes related to the DNA replication and metabolism

The DNA replication protein encoded by ORF 1, classified to the DNA replication and metabolism module, had a helix-turn-helix domain, a common denominator in basal and specific transcription factors in bacterial cells. They have been recruited to a wide range of functions, not only transcription regulation and DNA repair and replication, but also RNA metabolism, and protein-protein interactions [49]. KilA-N domain-containing protein (ORF 14) was a novel, conserved DNA-binding domain found at the N-terminus of the poxvirus D6R/NIR proteins, which may play a role as nuclease domains mediate additional and specific interactions with nucleic acids or proteins. Its homologs have been widely detected in large bacteria or eukaryotic DNA viruses and even in some protozoa and fungal DNA-binding APSES domains [50, 51].

### Recombination module in the genome of phage vB_OliS_GJ44

Tetra correlations between per 10 kb genome fragments of vB_OliS_GJ44 and its whole genome are shown in Fig. 3A. The high score demonstrates the higher adaptive ability of the genes to their genome. In the red elliptical part of Fig. 3A, which includes seven fragments (from 26th to 32th), the tetra correlations drop significantly, indicating that this sequence was less adapted to its genome. These seven fragments correspond to six recombinant genes and one AMG. AMG, which is a group of genes that can modulate host cell metabolism, has a closer relationship with the host genome [52]. The 28th fragment (Fig. 3A) has the lowest tetranucleotide frequency correlation (0.83), further indicated that the recombination module was more closely related to the host.

The recombination module included six proteins. RusA can resolve Holliday intermediates and correct the defects in genetic recombination and DNA repair associated with the inactivation of RuvA, RuvB, or RuvC [53]. Following a previous report, the RecG pathway of junction resolution can be stimulated by the expression of RusA resolvase, whose gene resides on a cryptic prophage, such as prophage lambda [54]. The recombination enhancement function of RecA-dependent nuclease is a 21-kDa RecA-dependent HNH endonuclease that can be targeted to produce a double-strand break at any desired DNA sequence [55]. This gene was first reported in the genome of Escherichia phage P1, which is a prophage infection enterobacter. The unique signature of prophage P1 is the lysogenic strategy in the cell, which acts as a low copy of plasmid in the cell on its lysogenic stage [56]. Typically, both dsDNA and ssDNA could be bound by RecA-dependent nuclease, but will not produce cleavage to ssDNA. Cofactors or proteins, such as RecA, ATP, or Mg^2+^ are required for RecA-dependent nuclease degrading ssDNA [57]. The protein NinB is located in enterophage lambda, which is one of the components of NinR in ORF family recombinases of lambda, specifically binding to ssDNA [58]. The YqaJ viral recombinase protein family might play a similar role to exonuclease in enterophage lambda, that integrases to the chromosome of the host through recombination and which have been demonstrated to have a crucial role in viral replication. The ERF family protein was first reported in Salmonella phage P22, which also promotes homologous recombination like the Red system in phage lambda [59, 60]. ERF protein has been commonly observed in temperate bacteriophages infecting *Gammaproteobacteria*, and could promote circularization of the linear dsDNA viral genome upon entry into the host cell [61, 62]. The combination module carried by vB_OliS_GJ44 could play a vital role in its replication in a host cell. The ssDNA-binding protein located in this module might interact with multiple recombination genes, as RusA family crossover junction endodeoxyribonuclease, protein NinB, Yqajdomain-containing exonuclease, and ERF family protein could act on ssDNA under certain conditions. Many genes within this module might play a similar role to the recombination process in phage lambda. However, there has been no integrase annotated for vB_OliS_GJ44, and a homolog of recombinase ORF of phage lambda [63] was not observed in the genome of vB_OliS_GJ44. Also unexpected was the presence of two phage antirepressor proteins (ORF 7 and ORF 60) “Phage antirepressor KilAC domain-containing protein”, which prevents the repressor protein of the P22 434 and lambda-like moderate prophage from binding to its operators, turn on the transcription of phage genes and promote propagation [64, 65]. This indicates that vB_OliS_GJ44 has a different strategy from the mild lambda-like phages. Given this, the propagation pathway in its host bacterium is unclear; the mechanism of recombination and propagation in vB_OliS_GJ44 requires further in-depth study.

### Tail-related genes of phage vB_OliS_GJ44

Compared with other siphoviruses that can infect *Oceanospirillaceae* (Marinomonas phage CPP1m 3, Marinomonas phage CB5A 3, Nitrincola phage 1 M3–16 3, Marinomonas phage P12026 1, and Marinomonas phage CPG1g 3), the number of tail-related proteins of vB_OliS_GJ44 was surprisingly high. A total of 13 genes were determined to be tail-related or cell adsorption and recognition proteins after PSI-BLAST analysis of all structural genes. The green line on the second circle of the genemap, accounting for 33% of the coding region (10,218 bp/31152 bp), represented the length of this region. These genes are tightly assembled into a continuous cluster.

ORF 29 was homologous with gene transfer agent family protein of *Bordetella genomo* sp. 7 with 98% coverage and 33% amino acid identity. In PSI-BLAST, most hits are of bacterial GTA (Gene transfer agent) proteins, which are derived from bacteria and archaea and are used to regulate horizontal gene transfer [66]. They are virus-like particles containing DNA fragments that can escape from mother cells and adhere to other cells to inject their DNA into the cytoplasm [67]. ORF 29 also hit tail protein sequences, except GTA protein in PSI-BLAST; it was speculated that ORF 29 mainly functions as a tail component in bacteriophages and identifying host cells.

ORF32 was annotated as a discoidin domain-containing protein, and homologues of this protein are widespread in bacteria proteins rather than phages in the NR database and usually play a role in cell adsorption. Discoidin domain-containing protein is a type of lectin, with an for galactose, that mediates cell adhesion and migration in the slime mould *Dictostelial discoideum* [68, 69]. The DS domain receptor family where the discoidin domain has usually been detected is in the cell outer membrane. It can bind to lipids such as glycans, polysaccharides, and collagens to regulates cell adhesion [70]. This protein is present in the phage genome and located in the tail protein cluster, so it may be related to the recognition of the receptor protein on the surface of the host cell.

The tails of siphoviruses are very efficient nanomachines, designed to infect the host, with extremely high specificity and effectiveness. They are essential for recognizing, attaching and piercing the host cell wall to ensure efficient delivery of genomic DNA to the host cytoplasm and determine the phage-specific characteristics, such as host range strategies [71]. The rich and diverse tail-related genes in the vB_OliS_GJ44 genome play an important role in the formation of the tail structure and the interaction between hosts.

### AMG and lysis genes

The only AMG in the whole genome vB_OliS_GJ44 is the MazG-like family protein (ORF 44), which regulates host cell metabolism and promotes infection efficiency during the process of bacteriophage infection of the host, is found in bacteriophages but originated from bacteria [50]. Similarity has been observed with the dimeric 2-deoxyuridine 5′-triphosphate nucleotidohydrolase (dUTP pyrophosphatase or dUTPase) and NTP-PPase MazG proteins. However, members of this family consist of a single MazG-like domain that contains a well-conserved divalent ion-binding motif EXXE/D, different from the typical tandem-domain MazG proteins [72]. Studies have suggested that the viral MazG protein may reduce the content of guanosine 3′,5′-bispyrophosphate (ppGpp) in the host, deceive the host into maintaining a ‘hungry state’, and accelerate the metabolism of the host bacteria to promote their reproduction [73–75]. However, given that the gene of NTP pyrophosphohydrolase was located in the recombination module, it my alternatively have some unknown function in the recombination progress of vB_OliS_GJ44, which requires further investigation.

The genome also encoded N-acetylmuramoyl-L-alanine amidase, a phage lysin, which catalyzes the chemical bond between N-acetylmuramoyl residues and L-alanine residues in cell wall glycopeptides [76], has been shown to be highly similar to the same protein predicted in a Marinomonas phage P12026 genome [77]. Both the genera *Marinomonas* and *Oceanospirillum* are classified into the family *Oceanospiraceae*. The TMhelix containing protein, which is close behind in the genome encoded by ORF 17 homologated with Vibrio phages of Autographiviridae, is related to the transport of substances across cell membranes [78] and may be related to infecting and lysing host.

### Phylogenetic analysis suggested that vB_OliS_GJ44 represents a novel viral cluster

**Fig. 4:**
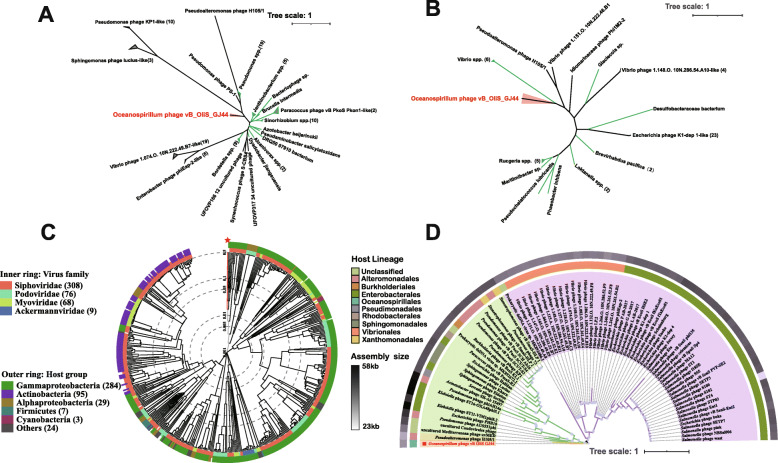
Phylogenetic trees of vB_OliS_GJ44 based on three different methods. **A**, **B** Unrooted maximum-likelihood dendrogram derived from amino acid sequences of the phage major capsid protein. and terminase large subunit respectively. The green branches represent that the protein sequences are from bacteria, and the black branches are from phages. **C** Phylogenetic analysis with other related phages identified using the genome-wide sequence similarity values computed by tBLASTx. **D** Maximum-likelihood phylogenetic tree of the vB_OliS_GJ44 inferred from a concatenated protein alignment of three hallmark proteins (MCP, TerL, and portal protein). Four shades of different colors indicate the boundaries of clades. Tree annotations from inside to the outside: (1) host lineage (2) assembly size

To further understand the phylogenetic relationship of vB_OliS_GJ44 to other isolated phages, three different types of phylogenetic trees were generated: single-gene, multi-genes, and whole-genome. MCP and TerL phylogenetic trees were established using 98 and 50 sequences respectively, with the highest homology through BLASTp against the NR database. In the MCP tree, (Fig. 4A) 53 homologous bacterial sequences and 45 virus sequences were selected, vB_OliS_GJ44 presents a separate branch and is far from the other sequences. Twenty homologous bacterial sequences and 30 phage sequences were constructed by the TerL tree (Fig. 4B), although vB_OliS_GJ44 is grouped with some *Vibrio* protein sequences, the branch lengths are 0.35 and 0.75, respectively, so the evolutionary distances are also relatively far.

Seventy-six viruses were selected according to the sequence similarity and their MCP, TerL-related genes and portal protein were connected in series to establish a multi-genes phylogenetic tree. Among them, vB_OliS_GJ44 originates from the tree root and forms a separate clade (Fig. 4D). In the phylogenetic tree based on 461 viral whole-genomes, nine siphoviruses are clustered together, but the branch length was about 0.48; this further demonstrates that vB_OliS_GJ44 represents a novel siphoviral cluster (Fig. 4C).

These results show that the identification of vB_OliS_GJ44 not only expands the catalog of marine Oceanospirillum phages but also represents a new cluster of marine phages. As the first isolation of a phage from genus *Oceanospirillum* and classified into a novel viral cluster, we propose that vB_OliS_GJ44 represents a novel viral genus, named *Oceanospirivirus*, in the *Siphoviridae*.

### The relationship between vB_OliS_GJ44 and uncultured phage contigs

During the last decade, through the application of metagenomics understanding of viral diversity has expanded rapidly, identifying 195,728 viral taxa from the global ocean. The was accomplished through a combination of isolation and genomic analyses, especially from the dominant and important bacterial groups, such as *Synechococcus*, *Roseobacter*, *Pseudoalteromonas*, *Alteromonas,* and *Vibrio* from coastal areas and *Pelagibacter* (SAR 11), *Puniceispirillum* (SAR 116) and *Prochlorococcus* from the open ocean [46, 79, 80].

**Fig. 5:**
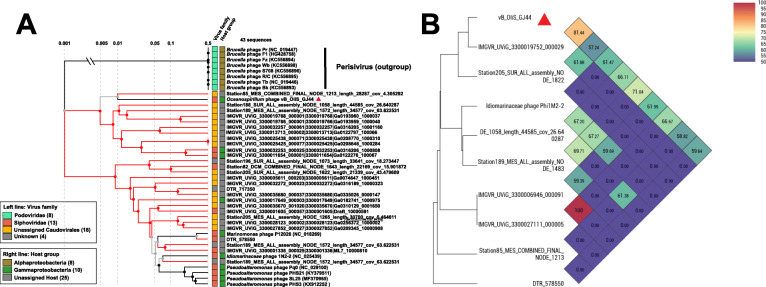
Comparisons of vB_OliS_GJ44 with other related uncultured phages in IMG/VR database. **A** Phylogenetic analysis with other related uncultured phages in IMG/VR database using the genome-wide sequence similarity values computed by tBLASTx. **B** Heat map based on OrthoANI values calculated using OAT software

vB_OliS_GJ44 lacks an obvious connection with the isolated virus strains in the NCBI virus database, perhaps because only a few phage isolates infect *Oceanospirillum*. Therefore, tBLASTx was used to search the IMG/VR [41] database in an attempt to expand the *Oceanospirivirus* database. The virus sequences in the IMG/VR [41] database is all derived from the assembly of metagenomic data. In total, 27 uncultured viruses were screened with at least 7 common genes. Thirteen isolated viruses together with vB_OliS_GJ44 were added to construct a genome-level phylogenetic tree. Phage vB_OliS_GJ44 and its closest relative, Station85_MES_COMBINED_FINAL_NODE_1213 (Station85_1213), are grouped into a diverse clade containing ten other marine phages, which shared the same node (Fig. 5A).

The Bacterial and Archaeal Viruses Subcommittee (BAVS) of the International Committee on the Taxonomy of Viruses (ICTV) considers phages sharing ≥50% nucleotide sequence identity as members of the same genus [81]. In Fig. 5B, the highest average nucleotide identity (ANI, 81.44%) was between vB_OliS_GJ44 and IMGVR_UViG_3300019752_000029, and the smallest ANI is 58.82% with Station85_1213. This result provided further support for the suggestion that vB_OliS_GJ44 and the uncultured page contigs may represent a new cluster genus, *Oceanospirivirus*, which is likely to be widely distributed.

### Comparative genomic analysis between vB_OliS_GJ44 and uncultured phages

**Fig. 6:**
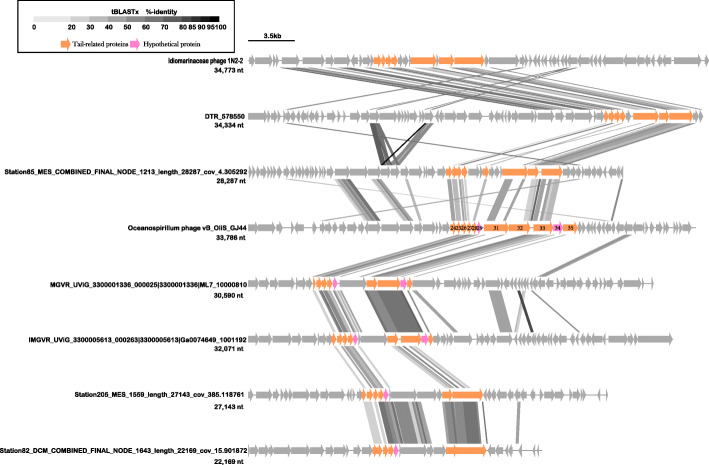
Comparative genomic analysis of the tail-related genes cassette of vB_OliS_GJ44 and other uncultured phage contigs. Sequence comparisons performed using tBLASTx (10 bp minimum alignment) with percent identity shown as a black box (inset scale bar). Synteny was recognized when genomes featured a minimum of five consecutive syntenic genes within the same genomic area and separated by a maximum of four non syntenic genes

In the comparative genomic analysis, vB_OliS_GJ44 showed some similarities to six uncultured phage contigs, most of which had similar genes that were continuous and concentrated in tail-related genes (Fig. 6). It is common to find some homologous genes encoding viral structural proteins among different *Caudovirales* genomes [82–85]. Tail-related genes are essential to the tail-phages for host adsorption and DNA ejection through the baseplate and the most effective gene arrangements. Unexpectedly, although synchronization was observed in all of the genome from tail-related virion proteins (ORF 24) to tail fiber proteins (ORF 35) (Fig. 6), there was almost no synchronization in other regions, except for Station85_1213, which had some synchronicity in the upstream area (terminase, MCP, and capsid assembly scaffolding protein) of the tail-related genes cassette. This lack of synchronization is probably due to the high genetic variability between these host recognition proteins. Indeed, a high level of variability among tail fibers has been reported several times [86].

### Distribution of ORF homologues of vB_OliS_GJ44 in marine viromes

Predicted ORFs from vB_OliS_GJ44 genomes were used to estimate relative abundances in quantitative POV, GOS, Malaspina viral metagenomes using a reciprocal best-BLAST approach with minor modifications. A total of 433 reads were successfully recruited at rates of approximately 10^− 10^ per amino acid pair in all three databases. In contrast, the ORF abundance was higher in GOS-estuary and POV-coastal areas, with 1.19E-07 and 1.33E-07 assigned reads per amino acid pair respectively (Table 2). Metagenomic analysis indicated that vB_OliS_GJ44 might be widely distributed in the ocean with low relative abundance. The relative abundances of vB_OliS_GJ44 in four viral metagenomes collected from the bathypelagic zone, > 4000 m, during the Malaspina Expedition (2011) were also investigated. Data shows that the abundance of phages in deep water is stable at 10^− 9^ reads per pair of amino acids.

**Table 2:** Recruitment detailed of Oceanospirillum phage vB_OliS_GJ44 ORFs against metagenomic databases

Virome	Number of virome reads	Number of recruitment sequence	Number of assigned reads per amino acid pair	vB_OliS_GJ44 ORF coverage
^a^GOS_coastal	3,246,085	697	2.59E-08	33.33%
^b^GOS_estuary	322,738	318	1.19E-07	18.33%
^c^GOS_open	3,272,816	224	8.25E-09	21.67%
^d^POV^−^coast	3,069,557	3385	1.33E-07	61.67%
^e^POV-intermediate	589,546	472	9.65E-08	33.33%
^f^POV-open	1,579,556	1174	8.96E-08	43.33%
^h^Malaspina_91	16,565,792	1180	8.59E-09	18.33%
^h^Malaspina_103	18,360,846	1885	1.24E-08	21.67%
^h^Malaspina_109	18,875,232	1065	6.80E-09	20.00%
^h^Malaspina_112	26,047,114	1550	7.17E-09	21.67%
^h^Malaspina_131	24,769,072	1603	7.80E-09	23.33%
^h^Malaspina_144	27,758,092	1200	5.21E-09	15.00%

^a^The Global Ocean Sampling (GOS) estuary database includes metagenomic data obtained from sampling sites GS006, GS011, GS012 and MOVE858 [44]

^b^The GOS coastal region database includes metagenomic data obtained from sites GS002, GS003, GS004, GS007, GS008, GS009, GS010, GS013, GS014, GS015, GS016, GS019, GS021, GS027, GS028, GS029, GS031, GS034, GS035, GS036, GS049, GS117a and GS149 [44]

^c^The GOS open ocean database includes metagenomic data obtained from sites GS017, GS018, GS022, GS023, GS026, GS037, GS047, GS109, GS110a, GS111, GS112, GS113, GS114, GS115, GS116, GS119, GS120, GS121, GS122 a, GS122b and GS123, according to Rusch et al. [44]

^d^The Pacific Ocean Virome (POV) coastal region dataset includes metagenomic data obtained from sampling sites 002255 M.Fall.C.10 m, 002256SFC.Spr.C.10 m, 002257SFD.Spr.C.10 m, 002258SFS. Spr.C.10 m, 0022259STC.Spr.C.10 m, 002260SMS.Spr.C.10 m, and 002245 L.Spr.C.10 m [43]

^e^The Pacific Ocean Virome (POV) intermediate region dataset includes metagenomic data obtained from sampling sites 002230 L.Spr.I.10 m and 002253 M.Fall.I.10 m [43]

^f^The Pacific Ocean Virome (POV) open ocean dataset includes metagenomic data obtained from sampling sites 002234 L.Sum.O.10 m, 002238 L.Win.O.10 m, 002242 L.Spr.O.10 m, and 002249 M.Fall.O.10 m [43]

^h^The Malaspina Expedition (2011) metagenomic dataset includes MSP91, MSP103, MSP109, MSP112, MSP131, MSP144 [45]

**Fig. 7:**
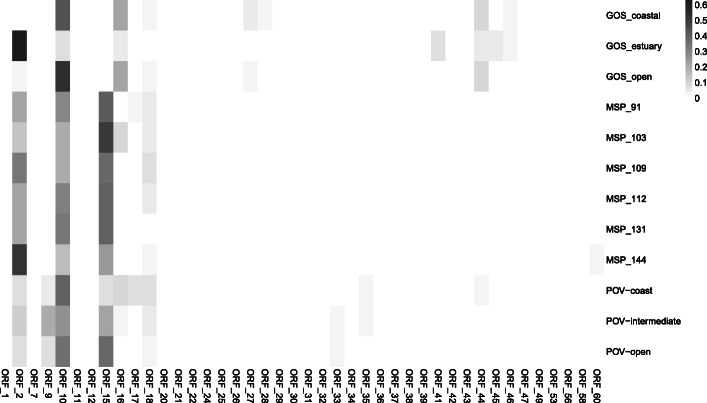
Relative abundance of homologs of vB_OliS_GJ44 phage genes in the metagenome datasets

Homologous sequences of each of the 60 ORFs were found in the database but the top five ORFs, with higher recruitment rates, were ORF 2 (AAA family ATPase, 2.84E-07 per pair), ORF 9 (DUF1289 domain-containing protein, 1.19E-07 per pair),

ORF 10 (N-acetylmuramoyl-L-alanine amidase, 4.68E-07 per pair), ORF 15 (Phage terminase small subunit, 2.90E-07 per pair), ORF 16 (Putative large terminase, 9.30E-08 per pair), which are mainly associated with phage replication, packaging, and lysis modules. A similar situation has also been found in other marine phages, such as Erythrobacter phage vB_EliS-R6L [87]. Several ORFs only have hits in a certain database, such as ORF39 (Hypothetical protein) and ORF53 (Hypothetical protein), that were only detected in the POV-open dataset. Similarly, ORF 22 (Hypothetical protein), ORF 25 (Putative head-tail joining protein), ORF 29 (Hypothetical protein), ORF 30 (Hypothetical protein), ORF 34 (Hypothetical protein), ORF 37 (Hypothetical protein), ORF 38 (Hypothetical protein), ORF 49 (Hypothetical protein), and ORF 58 (DNA-binding transcriptional regulator) were only detected in the POV-coast database (Fig. 7). The top five ORFs with the most recruitment were relatively abundant in each database. These results indicate that vB_OliS_GJ44 may represent a new and unknown ecological pedigree and provide a reference genome for the classification of environmental marine viral contigs in the future.

## Conclusions

*Oceanospirillum* has a very special niche and its phage will inevitably affect its community structure and metabolic efficiency. vB_OliS_GJ44 is the first isolated phage to infect *Oceanospirillum*. There are a large number of tail genes and a unique host-adapted recombination module in its genome architecture. Its evolutionary linage is novel and represents a cluster together with some uncultured virus sequences. This study has provided the first glimpse of the diversity, genomic evolution, abundance, and distribution of phages infecting *Oceanospirillum*. It provides a model interaction system and some new insights into interactions between *Oceanospirivirus* and Oceanspirillum phage-driven evolution and dynamics of their hosts, and the potential ecological significance of *Oceanospirivirus*. This study reinforces the importance of the combination of phage isolation and metagenomics to improve our knowledge of marine virus functions and diversity. Future isolation of phages infecting other *Oceanospirillum* species may disclose more novel phage clusters.

## Supplementary Information

**Additional file1: Table S1.** Genome annotation of phage vB_OliS_GJ44.

**Additional file 2.** 16S rRNA gene sequences of the bacteria used in host range test.

## Data Availability

The genome sequence of phage vB_OliS_GJ44 is available in the GenBank repository, https://www.ncbi.nlm.nih.gov/nuccore/MW560978 The 16S rRNA gene sequence of the host, *Oceanospirillum* sp. ZY01 is available in the GenBank repository, https://www.ncbi.nlm.nih.gov/nuccore/MW547060

## Abbreviations

% GC: Percent guanine: cytosine
qcov: Query coverage
AMG: Auxiliary metabolic gene
ANI: Average nucleotide identity
CDS: Coding DNA sequences
GOS: Global Ocean Sampling
GTA: Gene transfer agent
MCP: Major capsid protein
MOI: Optimal multiplicity of infection
NR: Nonredundant proteins
ORF: Open reading frames
PFU: Plaque-forming unit
POV: Pacific Ocean Virome
PSI-BLAST: Position-Specific Iterated BLAST
RBB: Reciprocal best-hit BLASTp
TEM: Transmission electron microscopy
TerL: Terminase large subunit
Malaspina_: Malaspina viral metagenomes
PFU: Plaque forming unit
CFU: Colony-forming unit
